# Pollen, Tapetum, and Orbicule Development in *Colletia paradoxa* and *Discaria americana* (Rhamnaceae)

**DOI:** 10.1100/2012/948469

**Published:** 2012-05-02

**Authors:** M. Gotelli, B. Galati, D. Medan

**Affiliations:** Grupo de Biología Reproductiva en Plantas Superiores, Cátedra de Botánica Agrícola, Facultad de Agronomía, Universidad de Buenos Aires, Avenida San Martín 4453, 1417 Buenos Aires, Argentina

## Abstract

Tapetum, orbicule, and pollen grain ontogeny in *Colletia paradoxa* and *Discaria americana* were studied with transmission electron microscopy (TEM). The ultrastructural changes observed during the different stages of development in the tapetal cells and related to orbicule and pollen grain formation are described. The proorbicules have the appearance of lipid globule, and their formation is related to the endoplasmic reticulum of rough type (ERr). This is the first report on the presence of orbicules in the family Rhamnaceae. Pollen grains are shed at the bicellular stage.

## 1. Introduction

Rhamnaceae is a family of about 55 genera and 900 species, cosmopolitan in distribution, especially in warm temperate regions [1, 2]. The tribe Colletieae is a monophyletic group that comprises six genera that differ in flower and fruit traits [3–7]. Distribution of the tribe is associated with the Andes in South America, and usually found 30° South [7]. The traditional diagnostic characters of the tribe are decussate leaves, abundance of spines, and presence of serial meristems in the leaf axils [8].

Literature on pollen morphology of the Rhamnaceae is relatively abundant, and descriptions are often given [2, 9–11]. However, ultrastructural studies on the development of pollen grains in Rhamnaceae are rare and restricted to descriptions of the pollen grain wall. Microsporogenesis, microgametogenesis, and the sporophytic structures related, such as the tapetum, are not usually considered [12].

The aim of this paper is to describe the ultrastructure of pollen grains and microsporangium development of *Colletia paradoxa *and *Discaria americana *in order to broaden the current embryological knowledge of the Rhamnaceae family.

## 2. Materials and Methods

Samples of *Colletia paradoxa* and *Discaria americana* were collected from individuals cultivated in the Lucien Hauman Botanical Garden of the Facultad de Agronomía, Universidad de Buenos Aires. Reference specimens were deposited in the Herbarium Gaspar Xuarez (BAA).

For transmission electron microscopy, anthers at different developmental stages were prefixed overnight in 2.5% glutaraldehyde in phosphate buffer (pH 7.2) and then postfixed in OsO_4_ at 2°C in the same buffer for 3 h. Following dehydration in ethanol series, the material was embedded in Spurr's resin. Ultrathin sections (750 to 900 nm) were made on a Sorvall ultramicrotome and then stained with uranyl acetate and lead citrate [13]. The sections were observed and photographed with a JEOL-JEM 1200 EX II TEM at 85.0 kV.

Resistant membranes with orbicules and pollen walls were isolated by acetolysis of whole anthers. The acetolysis was carried out following Erdtman's method [14]. Acetolysis-resistant structures were washed with water and mounted in glycerin-gelatin.

## 3. Results

### 3.1. Stage 1: Microspore Mother Cells (MMCs)

**Figure 1:**
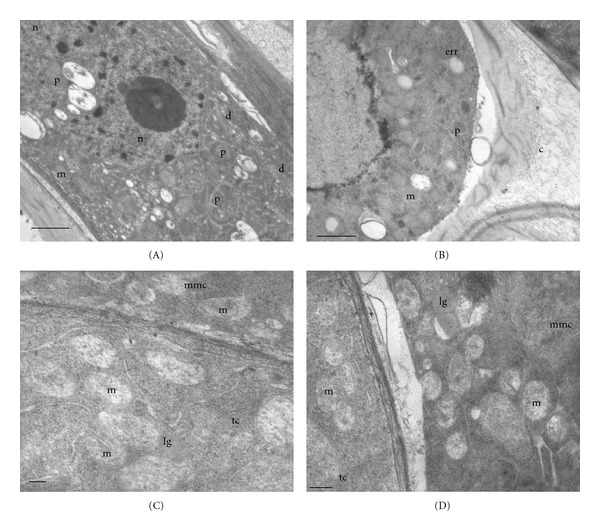
Stage 1: microspore mother cells (MMCs). (A)-(B) *Colletia paradoxa.* (C)-(D) *Discaria americana*. (A) Tapetal cell with two nuclei (n), plastids (p), dictyosomes (d), and mitochondria (m). (B) Details of the microspore mother cell: plastids (p), mitochondria (m), endoplasmic reticulum of rough type (err), and callose (c). (C) Microspore mother cell (mmc) and tapetal cell (tc) with mitochondria (m) and lipid globules (lg) (D) Microspore mother cell with lipid globules (lg). and mitochondria (m). Scale bar; (A)-(B) 1.3 *μ*; (C) 200 nm (D) 500 nm.

In both species, the anther is bi-sporangiate and its wall consists of epidermis (ep), endothecium (en), two or three middle layers (ml), and a secretory type tapetum (t). Tapetal cells are binucleate. They present a cytoplasm with a few small vacuoles, mitochondria, and many dictyosomes and proplastids (Figure  1(A)) in *Colletia paradoxa*, and mitochondria and lipid globules in *Discaria americana* (Figure  1(B)).

The microspore mother cells are uninucleate and with a dense cytoplasm. In *Colletia paradoxa*, it is filled with abundant mitochondria, plastids, and endoplasmatic reticulum of rough type (ERR). A thick callosic wall is formed between the plasmalemma and the primary wall (Figure  1(C)). In *Discaria americana*, lipid globules and mitochondria are observed (Figure  1(D)).

### 3.2. Stage 2: Microspore Tetrads

**Figure 2:**
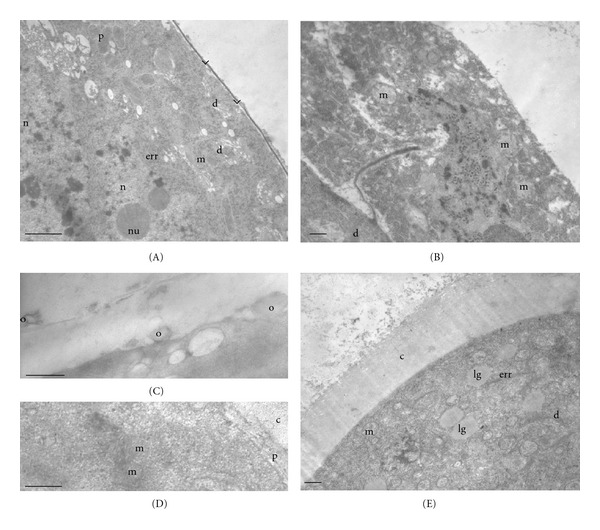
Stage 2: microspore tetrads. (A)–(C) *Colletia paradoxa.* (D)-(E) *Discaria americana.* (A) Tapetal cell at young microspore tetrad stage. Proorbicules (arrow head) are observed. Cytoplasm with endoplasmic reticulum of rough type (err), plastids (p), mitochondria (m), dictyosomes (d), and two nuclei (n), and nucleoli (nu). (B) Details of the tapetal cytoplasm and wall at an advanced microspore tetrad stage. Orbicules (o) are observed in the inner and the outer tapetal wall, which is partly degraded. Dictyosomes (d) and mitochondria (m) are also present. (C) Microspore with many mitochondria (m) and dictyosome (d). Callosic wall and primexine are observed. (D) Tapetal cell with many mitochondria (m) and dictyosomes (d). Cell wall degraded. (E) Microspore with mitochondria (m), lipid globules (lg), dictyosomes (d), endoplasmic reticulum of rough type (err), and callose (c). Scale bar: (A) 1.3 *μ*; (B) 300 nm; (C) 400 nm; (D)-(E) 500 nm.

The ultrastructure of the cytoplasm of tapetal cells of *Colletia paradoxa* is similar to that presented in the previous stage. Many dictyosomes, mitochondria, and plastids are present along with some endoplasmic reticulum of rough type (Figure  2(A)). In the inner tangential faces of these cells, proorbicules are observed as globular depositions of moderate electron density between the plasmalemma and the cell wall (Figure  2(A)). *Discaria americana* presents the cytoplasm of tapetal cells very dense with many mitochondria, and the cell wall is no longer distinguished in this stage (Figure  2(B)).

Microspore mother cells undergo simultaneous meiosis, forming tetrads with a tetrahedral arrangement. They remain surrounded by a thick callosic wall. At this stage, the primexine fibrillar matrix is starting to develop between the callosic wall and the plasmalemma (Figure  2(D)). The microspore cytoplasm shows many mitochondria and a few dictyosomes in *Colletia paradoxa* (Figure  2(D)), while in *Discaria americana* mitochondria, dictyosomes, endoplasmic reticulum of rough type, and lipid globules are observed (Figure  2(E)).

As the microspore tetrad matures, more mitochondria are found in the cytoplasm of tapetal cells of *Colletia paradoxa*. The wall of these cells appears laxer and degraded in some places. Orbicules are released and some of them are observed on the outer tangential cell wall of the tapetal cells (Figure  2(C)). An electron-dense deposition is observed on the surface of the proorbicules (Figure  2(C)). 

### 3.3. Stage 3: Free Microspores

**Figure 3:**
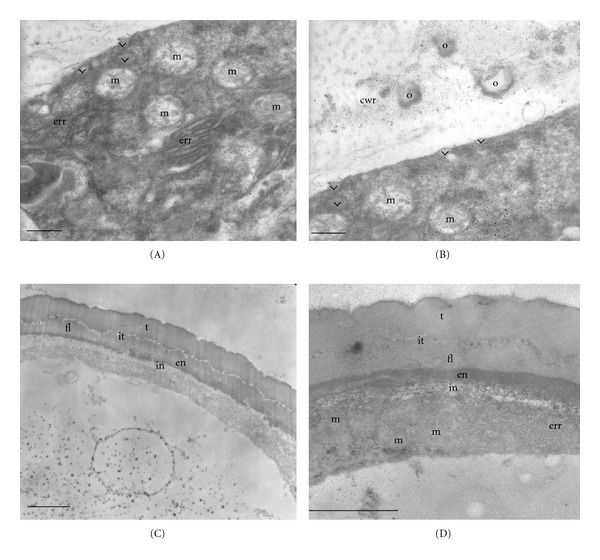
Stage 3: free microspores. *Colletia paradoxa.* (A) Tapetal cell with proorbicules (arrow head), many mitochondria (m), and rough endoplamic reticulum (err). (B) Details of tapetal cell, wall degraded, and orbicules are observed between its remains (cwr: cell wall remains). Mitochondria (m) and proorbicules (arrow head) are also observed. (C) Young microspore: intine (in), endexine (en), foot layer (fl), a tectum (t), and a granular infratectum (it) are clearly distinguished. (D) Details of the young microspore cytoplasm with mitochondria (m) and endoplasmic reticulum of tough type (err). Scale bar: (A) 280 nm; (B) 300 nm; (C)-(D) 1.3 *μ*.

At this stage, the cell wall of tapetal cells in *Colletia paradoxa *is also dissolved and remains of it are observed between the orbicules, which have been released (Figure  3(B)). Many mitochondria and endoplasmatic reticulum of rough type are present in the cytoplasm of tapetal cells. There are vesicles near and in close contact to the plasmalemma (Figures  3(A)-3(B)). The ultrastructure of the cytoplasm of tapetal cells of *Discaria americana* is similar to that presented in the previous stage.

After the dissolution of the callose wall, the sporopollenin wall begins to form. The intine, endexine, foot layer, a tectum, and a granular infratectum are clearly distinguished in the two species (Figures  3(C)-3(D)). Microspores have a conspicuous nucleus, and their cytoplasm is limited to a parietal position due to the presence of a large vacuole. The structure of their cytoplasm is similar to the previous stage with many mitochondria and some endoplasmic reticulum of rough type (Figure  3(D)).

### 3.4. Stage 4: Young Pollen Grain

**Figure 4:**
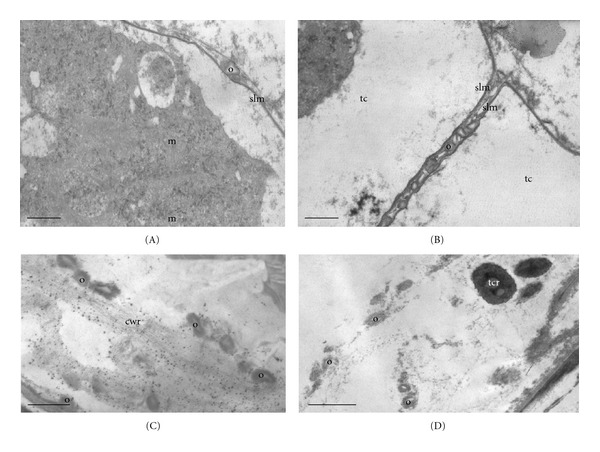
(A)-(B) Stage 4: young pollen grain. (C)-(D) Stage 5: mature pollen grain. (A)–(C) *Colletia *paradoxa. (D) *Discaria americana. *(A) Tapetal cell cytoplasm in degradation with some mitochondria (m) still recognizable and orbicules (o) attached to a sporopollenin-like. membrane (slm). (B) Orbicules (o) attached to the radial face of the sporopollenin-like membrane (slm) formed between two tapetal cells (tc) (C) Remains of the tapetal wall (cwr) with orbicules (o) and details of the sporopollenin-like membrane (slm) with orbicules (o) attached. (D) Orbicules (o) and tapetal cell remains (tcr). Scale bar: (A)-(B) 850 nm; (C) 500 nm; (D) 1 *μ*.

Tapetal cells are almost completely degraded in *Discaria americana* but in *Colletia paradoxa* do not lose their individuality. Some mitochondria and numerous free ribosomes can still be observed on their cytoplasm. However, most membrane systems appear to be disintegrating. In this species, a tapetal membrane is formed in the inner tangential and radial faces of tapetal cells and the orbicules are observed on it. This membrane is partially resistant to acetolysis because fragments of it are observed after this treatment. Orbicules present a central core transparent to electrons, and a wall with the same electron density that is the pollen exine. They have a spherical to subspherical shape with a few irregularities and superficial invaginations in both species (Figures  4(A)-4(B)).

**Figure 5:**
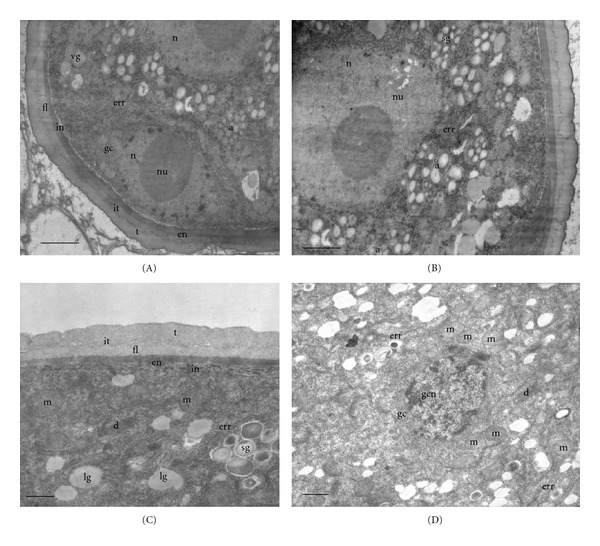
(A)-(B) Stage 4: young pollen grain of* Colletia paradoxa.* (C)-(D) Stage 5: mature pollen grain of* Discaria americana.* (A) Vegetative cell (VC), with endoplasmic reticulum of rough type (err), amyloplasts (a), and its nucleus (n) and nucleolus (nu); generative cell (GC) with its nucleus (n) and nucleolus (nu); tapetal cells (tc) and orbicules (o). (B) Details of the vegetative cell. (C) Generative cell with starch grains (sg), lipid globules, mitochondria (m), dictyosomes (d), endoplasmic reticulum of rough type (err). (D) Generative cell (gc) and its nucleus (gcn). Other references: t: tectum, it: infratectum, ft: foot layer, en: endexine, in: intine. Scale bar: (A)-(B) 1.3 *μ*; (C) 500 nm; (D) 1 *μ*.

The generative cell formed by a mitotic division of the microspore occupies a parietal position. A conspicuous nucleus occupies almost all the cytoplasm. Some mitochondria can be observed. It is delimited by a thin wall transparent to electrons and shows a few connections with the vegetative cell, which has a very dense cytoplasm filled with amyloplasts and endoplasmic reticulum of rough type (Figures  5(A)-5(B)).

### 3.5. Stage 5: Mature Pollen Grain

At this stage, tapetal cells cytoplasm appears almost completely degraded in both species. The tapetal membrane can still be observed with some orbicules attached to it in *Colletia paradoxa.* Orbicules are also attached to the remains of the original tapetal cell wall, which presents a fibrillar aspect (Figure  4(C)). Remains of these cells in *Discaria americana* appear as electron-dense rests between the orbicules. The average size of orbicules in both species is 0.4 *μ*m (Figure  4(D)).

The vegetative cell of the mature pollen grain presents many mitochondria, dictyosomes, starch grains, lipid globules, and endoplasmic reticulum of rough type (Figure  5(C)). The generative cell occupies a central position and has a reduced cytoplasm and cell wall practically absent only represented by the two adjacent membranes (Figure  5(D)).

**Figure 6:**
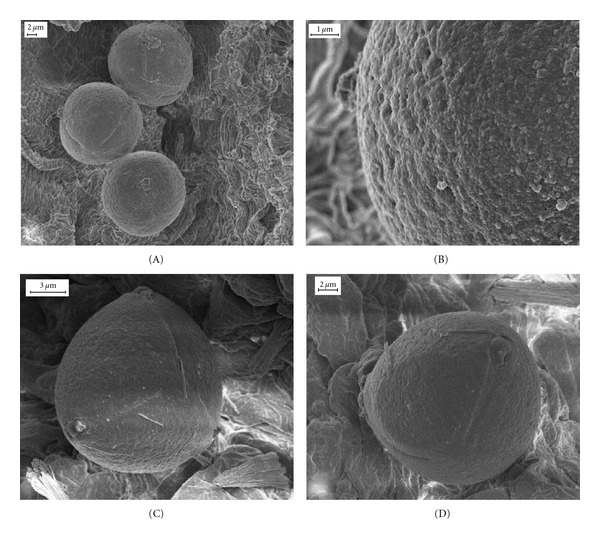
Stage 5: mature pollen grains. (A)-(B) *Colletia paradoxa*. (C)-(D) *Discaria americana*. (A) General view of pollen grains. (B) Details of a pollen grain. (C) Polar view of a pollen grain. (D) Equatorial view of a pollen grain.

The pollen grain wall is formed by three layers with different electron density: ectexine with a perforated tectum, a granular infratectum, and a foot layer thinner than the tectum; an electron-dense endexine; a thin intine of low electron density (Figures  5(A)–5(C)). Pollen grains of both species are suboblate, 3-colporate, tectum fossulate- perforate (Figures  6(A)–6(D)).

## 4. Discussion

Anther wall development in *Colletia paradoxa* and *Discaria americana* is of the basic type [15]. This feature was described as a general trait of the Rhamnaceae [16]. Medan and Aagesen [3] claim that in *Colletia paradoxa* there are often two or three pollen sacs in each anther, as here observed. The anther wall of both species comprises the epidermis, fibrous endothecium, two or three middle layers, and a secretory tapetum with binucleate cells as described for other members of the family [17]. The third middle layer is originated by the division of any of the first two middle layers. Cytokinesis is simultaneous, and the arrangement of microspores in the tetrad is tetrahedral for both species. Tetrahedral and isobilateral tetrads were reported to appear in the family [17].

Orbicules are corpuscles of sporopollenin lining in the inner tangential and sometimes in the radial tapetal cell walls that appear during pollen grain development [18, 19]. Ubisch [20] gave a detailed description of them. Kosmath [21] called the Ubisch bodies and Erdtman et al. [22] orbicules. Its size is very variable, ranging from 0.14 *μ*m to 20 *μ*m [19]. The average size in the species studied is 0.40 *μ*m. Huysmans et al. [18] studied the distribution of orbicules in Angiosperms, and there is no report of their presence in Rhamnales. This is the first report of orbicules in this taxonomic group.

Orbicules of *Colletia paradoxa* and *Discaria americana *have a central core transparent to electrons and a sporopollenin wall with a spheric to subspheric shape, with a few irregularities and superficial invaginations. This type of orbicule was previously described in the classification given by Galati [23]. The central core of orbicules in these two species appears as a proorbicule at the microspore tetrad stage. According to Clément and Audran [24] the orbicule core represents remnants of the proorbicule and plays an important role in the orbicular wall ontogenesis. A fibrillar substance with the same electron density as sporopollenin accumulates on the proorbicules after they are released. Therefore, the accretion of sporopollenin on them appears extracellular. This is in concordance with Echlin [25], Christensen et al. [26], Clément and Audran [24], Galati and Rosenfeldt [27], Galati and Strittmatter [28], Amela García et al. [29], and Rosenfeldt and Galati [30].

In *Colletia paradoxa*, proorbicules are observed in the tapetal cells at an early microspore tetrad stage. Later on this stage, orbicules are released and pass through the degraded zones of the tapetal cell wall and some of them are observed at the outer tangential wall of tapetal cells. At the young microspore stage, tapetal cell wall starts to disintegrate and orbicules are observed between its remains. Therefore, orbicules are fully developed and become in contact with the locular fluid even before the complete loss of the tapetal cell wall. In *Jacaranda mimosifolia* [28] and *Pinus sylvestris* [31, 32] tapetal cell walls present a lax structure and orbicules are observed protruding it.

An increase in the amount of ERR in the cytoplasm of tapetal cells of *Colletia paradoxa* was observed at the young microspore stage, when most orbicules are being released. ERR is usually found close to the inner tangential wall, and little vesicles similar to proorbicules are in association with it. This suggests that this organelle is related with the formation of these orbicules, as observed in *Ceiba insignis* [27], *Jacaranda mimosifolia* [28], *Prosopis juliflora* [33], *Catharanthus roseus* [34], *Anemarrhena asphodeloides* [35], *Lilium henryi* [36], *Lavandula dentata* [37], and *Allium cepa* [38].

At the young pollen grain stage, orbicules in *Discaria americana* appear lose in the anther locule, while in *Colletia paradoxa* they are attached to a tapetal membrane as observed in *Jacaranda mimosifolia* [28]. The presence of orbicules is generally associated with a tapetal membrane in species with secretor type tapetum and with a peritapetal membrane in species with intermediate or plasmodial type tapetum [19]. Tapetal membranes are usually described as tapetally secreted sporopollenin membrane that develops on the inner tangential face of the tapetum [39]. In *Colletia paradoxa*, such structure is considered a membrane because it is partially resistant to acetolysis and also develops on the radial faces of tapetal cells. Therefore, orbicules are observed in the inner tangential and radial faces of this membrane.

In *Jacaranda mimosifolia*, the loss of tapetal cell walls occurs at the bicellular pollen grain stage, simultaneously with the formation of the tapetal membrane [28]. In *Colletia paradoxa*, tapetal cell walls start to degrade at the mature microspore tetrad stage. However, the sporopollenin-like tapetal membrane is formed at the young pollen grain stage. In *Colletia paradoxa*, tapetal cells degrade at the mature pollen grain stage, possibly due to the presence of such membrane, while in *Discaria Americana*, where no membrane was observed, tapetal cells degrade in the free microspore stage.

Many speculations were made trying to understand the role of orbicules [26, 34, 36, 40, 41]. However, none of them could be confirmed. Heslop-Harrison [42, 43] was the first to consider that the orbicules could be associated with pollen dispersal forming a not-wettable surface from which pollen can easily be freed. Later, Keijzer [44] supported this theory. Pacini and Franchi [45] wondered if pollen grains and orbicules would repulse each other, since they are formed by the same substance and, therefore, charged in the same way. Vaknin et al. [46] analyzed the role of electrostatic forces. Galati et al. [19] related orbicule morphology of representative species of diverse families of Angiosperms with different modes of pollen dispersal concluding that families, which may be taxonomically distant but share the mode of pollen dispersal, present similar orbicule morphology. All species of Colletieae studied to date show pollen transfer mediated by insects (Diptera, Hymenoptera, and Lepidoptera) [7]. This mode of pollen dispersal was described in species with orbicules spheric to subspheric, with a central core and an irregular surface, as here described. Therefore, these findings support the theory proposed by Galati et al. [19].

Pollen grain morphology is in accordance with the general morphological pattern of the family Rhamnaceae [2, 11, 17, 47]. In *Ziziphus lotus* L., the exine is characterized by a thick tectum, a thin infratectal layer that appears granular, and a thick nexine [12]. Similar observations were made by Schirarend and Kohler [10] in *Colletia spinosissima.*

This is the first report of the ontogeny and ultrastructure of the pollen grain and related sporophytic structures of *Colletia paradoxa* and *Discaria americana*. Therefore, we consider it to be an important contribution to the general knowledge of the Rhamnaceae family.
